# Successful Desensitization to Irinotecan in a Patient with Metastatic Esophageal Squamous Cell Carcinoma and a History of Anaphylaxis in Response to Irinotecan—Case Report and Literature Review

**DOI:** 10.3390/jcm13247824

**Published:** 2024-12-21

**Authors:** Selda Ali, Denisa-Mihaela Nedelcu, Radu Serescu, Roxana Silvia Bumbăcea

**Affiliations:** 1 Allergology Department, “Carol Davila” University of Medicine and Pharmacy, 050474 Bucharest, Romaniaroxana.bumbacea@umfcd.ro (R.S.B.); 2 Allergology Department, “Dr. Carol Davila” Nephrology Clinical Hospital, 010731 Bucharest, Romania; 3 Amethyst Radiotherapy Center, 075100 Bucharest, Romania; raduserescu@gmail.com

**Keywords:** esophageal cancer, *irinotecan*, chemotherapy, anaphylaxis, skin test, desensitization protocol

## Abstract

**Background:** *Irinotecan* is a topoisomerase I inhibitor used for the treatment of various cancers, such as gastrointestinal, pancreatic, pulmonary, ovarian, and cervical cancers. Among chemotherapy agents, it represents a rare trigger of drug hypersensitivity reactions, with few cases being reported until today. **Methods:** We present the case of a patient with metastatic esophageal cancer and a history of *irinotecan*-induced grade IV (WAO classification) anaphylaxis. An IgE-mediated reaction was confirmed in our case, as evidenced by a positive intradermal skin test result, and we carried out a successful desensitization protocol, given *irinotecan’s* indispensability in the treatment regimen. Our case underscores the fact that in such situations where the culprit drug is also the only therapeutic option available for such a patient, implementing a desensitization protocol may represent the only viable approach to ensure safe and successful dosing. **Results:** A comprehensive review of the literature was also conducted to assess previously reported *irinotecan*-induced hypersensitivity reactions, the utility of skin tests in identifying sensitisation to *irinotecan*, and the existing desensitization protocols. We found a total of seventeen cases of hypersensitivity reactions to *irinotecan* in the literature, out of which four provided the skin test results obtained and six performed desensitization protocols for *irinotecan*. **Conclusions:** Our literature review highlights that skin testing and desensitization protocols can provide suitable solutions for managing hypersensitivity reactions to *irinotecan*.

## 1. Introduction

Chemotherapy agents currently play an important role in the treatment of various types of cancers and are widely used in clinical practice. However, they are not devoid of the risk of triggering hypersensitivity reactions. Platinum compounds, taxanes, epipodophyllotoxins, and *asparaginase* are the most frequent chemotherapy drugs known to elicit hypersensitivity reactions. Nonetheless, all chemotherapy treatments carry this risk [1]. Several diagnostic algorithms and management strategies for hypersensitivity reactions induced by chemotherapeutic agents have been proposed [2]. When hypersensitivity reactions occur and no equally effective alternative drug is available, desensitization procedures should be considered [3].

*Irinotecan* is a topoisomerase I inhibitor that is used in a variety of neoplasms, such as gastrointestinal, pancreatic, pulmonary, ovarian, and cervical cancers [4]. It has a complex pharmacology involving trapping topoisomerase I on DNA, inducing cytotoxic protein-linked DNA breaks [5]. Until now, it has been successfully used in different combinations with other chemotherapeutics (*cisplatin* or *oxaliplatin*) or tyrosine kinase inhibitors (*apatinib*, *dasatinib*, *lapatinib*, *pazopanib*, *regorafenib*, and *sunitinib*) for the treatment of malignancies. Genetic studies are currently underway to better define patients who benefit from the association of irinotecan with the aforementioned drugs, with an optimal risk/benefit ratio [6].

Even though hypersensitivity reactions to *irinotecan* are rare and the exact prevalence has not been established, a few cases have been reported, with four of them also having undergone desensitization protocols [7,8,9,10,11].

The desensitization process alters the immune system’s responsiveness to a medication, creating a transient tolerance that enables a patient who has experienced a drug hypersensitivity reaction to safely tolerate it [12]. The process involves administering the medication in increasing dosages until the prescribed level is achieved. If the drug is administered intermittently, tolerance is lost and a new desensitization process is necessary; this is also applicable for chemotherapy. The desensitization procedure does not lack risks, which is why it is recommended only if the treatment is essential and no other equally effective drug is available.

We present the case of a patient with metastatic esophageal cancer and a history of anaphylaxis in response to *irinotecan*, for whom a successful desensitization protocol was carried out. Similar to the cases reported in previous research, we achieved a safe and effective outcome by adapting the protocols used in the available literature to our situation. As *irinotecan* represents a rare trigger for hypersensitivity reactions, every case adds value to the literature and further increases knowledge about the clinical and paraclinical picture and management options.

## 2. Case Report

A 53-year-old female patient suffering from metastatic esophageal squamous cell carcinoma with a history of anaphylaxis in response to *irinotecan* was referred by her attending oncologist to our clinic for an allergy work-up in order to evaluate the possibility of continuing treatment with *irinotecan*.

In March 2021, the patient was diagnosed with stage II grade 3 (cT3cN0cM0) esophageal squamous cell carcinoma, at which point the presence of some isolated non-specific pulmonary micronodules (4 mm) was identified via CT scan.

The Oncology Tumor Board evaluated the patient and recommended treatment according to the CROSS (Chemoradiotherapy for Oesophageal Cancer Followed by Surgery) trial protocol. The regimen includes preoperative chemoradiotherapy delivering 45 Gy over 5 weeks, combined with weekly administration of *carboplatin* at an area under the curve (AUC) of 2 mg/mL·min and paclitaxel at a dose of 50 mg/m^2^. This regimen commenced on 12 April 2021 and concluded on 14 May 2021. A subsequent evaluation by the TB using a PET-CT scan on 7 July 2021 showed no progression of the disease, leading to the decision to perform an esophagectomy and an esophagoplasty with cervical esophagogastric anastomosis. The post-surgery histopathological findings identified ypT1b ypN1 (indicating perigastric lymph node micrometastasis). Therefore, adjuvant therapy with *nivolumab* was initially indicated for the patient; however, at that time, *nivolumab* was not reimbursed in Romania, hence, the patient did not receive this treatment. By December 2021, the patient had achieved a Disease-Free Survival (DFS) of five months post-surgery; however, a PET-CT scan indicated the presence of pulmonary nodules with a diameter of 1 cm, suggestive of metastasis.

Due to the non-reimbursement of first-line immunotherapy, the patient commenced chemotherapy exclusively, using the modified *Folinic acid*, *Fluorouracil*, *Oxaliplatin* (mFOLFOX) regimen, from December 2021 to October 2022. This was in conjunction with stereotactic body radiation therapy (SBRT) for pulmonary nodules, characteristic of oligometastatic disease, delivering a total dose of 48 Gy in four fractions of 12 Gy each. Subsequently, the patient transitioned to maintenance therapy with *Capecitabine* due to the development of second-grade neuropathy attributable to the toxicity of *Oxaliplatin*.

The patient was subjected to follow-up assessments with CT scans every 3 months; however, on 13 March 2023, a PET-CT scan was conducted, revealing disease progression according to the Positron Emission Tomography Response in Solid Tumors (PERCIST) criteria. This included the emergence of a new pulmonary nodule located in the left inferior segment, measuring 9 mm in diameter and with a standard uptake value (SUV) of 4.

The patient presented with neuropathy, and the multidisciplinary team decided that the treatment of choice consisted of the modified *Folinic acid*, *Fluorouracil*, *Irinotecan* (mFOLFIRI) regimen. This regimen included *irinotecan* administered at a dose of 90 mg/m^2^ per day over 90 min, followed by *calcium folinate* at 400 mg/m^2^ per day over 120 min via a port-a-cath. *Fluorouracil* was given at 2400 mg/m^2^ per day over 48 h through an ambulatory infusion pump connected to the port-a-cath. Each cycle of *irinotecan* consisted of two infusions administered 48 h apart, on days 1 and 3.

This regimen was administered every two weeks for five months. Subsequently, the patient underwent SBRT for limited oligoprogressive disease, targeting the pulmonary nodules. The radiotherapy treatment, administered in September 2023, included a dose of 48 Gy in 12 Gy fractions across four fractions and 56 Gy in 8 Gy fractions across seven fractions.

On the 25th of October 2023, after 7 months of treatment, during the 11th cycle, the patient experienced a hypersensitivity reaction shortly after the initiation of *irinotecan* administration at a dose of 120 mg. Within 5–10 min of the infusion’s onset, the patient developed angioedema of the lips and tongue, dysphagia, rhinorrhea, abdominal cramps, blurry vision, a 20 mmHg drop in blood pressure, and tachycardia. These symptoms were consistent with a grade IV anaphylactic reaction, according to the World Allergy Organization (WAO) classification [13]. The infusion was immediately halted, and the patient was administered treatment in accordance with the management protocols delineated in the WAO guidelines for anaphylaxis [13]. Following the reaction, the patient continued treatment with *fluorouracil* and *calcium folinate* alone.

Since *irinotecan* was already a second-line treatment, the attending oncologist referred the patient for an allergy work-up, declining other therapeutic options like taxanes because of the persistent neuropathy. Therefore, the patient was presented to our department in December 2023, two months after the reaction, for a complete allergy work-up and the initiation of a desensitization protocol for *irinotecan*.

Upon physical examination, the patient was underweight and presented surgical scars from the previous esophagectomy and esophagoplasty. She displayed signs of peripheral neuropathy, including diminished sensation and reduced reflexes in the distal extremities. From a cardiovascular perspective, the patient was stable, with normal vital signs.

**Figure 1:**
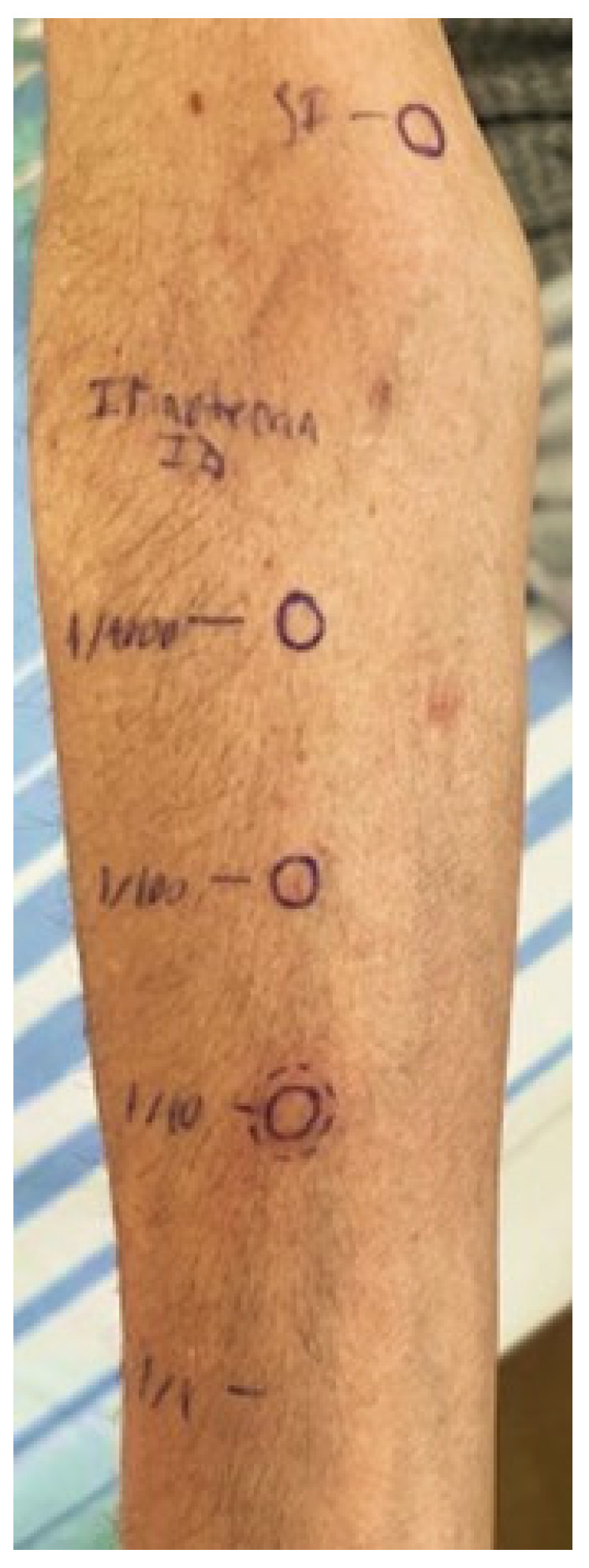
Positive response to *irinotecan* intradermal test, 2 mg/mL concentration.

To evaluate the hypersensitivity reaction, we decided to perform skin tests using *irinotecan*. The results of the skin tests and the concentrations used are summarized in Table 1. Due to the severity of the hypersensitivity reaction induced by *irinotecan*, we started performing the skin prick tests for *irinotecan* with a diluted solution (1/10 of the non-irritative concentration, 2 mg/mL). For the negative and positive controls, we used saline solution 0.9% and histamine 10 mg/mL, respectively. We performed the reading 15 min later, and we continued with the native solution at the maximum non-irritative concentration (20 mg/mL). Given the negative results of the skin prick tests, we performed intradermal tests, beginning with 1/100 (0.02 mg/mL) of the maximum non-irritative concentration, followed by the 1/10 dilution at 2 mg/mL. The intradermal test using a concentration of 2 mg/mL (1/10 dilution) revealed a 3 mm increase in wheal size accompanied by surrounding erythema, indicative of a positive intradermal test, as shown in Figure 1. Thus, an IgE-mediated sensitization to *irinotecan* was confirmed.

The following day, the desensitization protocol was carried out. The protocol was adapted from that of Castells et al. [14]. Premedication was administered 2 h before the initiation of the protocol, namely Dexamethasone 8 mg i.v, Rupatadine 10 mg, and Montelukast 10 mg. *Irinotecan* (20 mg/mL) was diluted in saline solution into three different concentrations; solution 1 (0.005 mg/mL), solution 2 (0.05 mg/mL), and solution 3 (0.5 mg/mL), which were progressively administered through a syringe pump in 12 steps. The protocol is depicted in Table 2. The rate of the infusion was increased every 15 min, and after the first hour, we changed the solution, using the next concentration, repeating the same operation over the following hour. As the attending oncologist recommended, a cumulative dose of 120 mg of irinotecan was reached over approximately 6 h. The desensitization protocol was well tolerated by the patient, without any sign of an immediate hypersensitivity reaction. The patient was discharged the following day in good general condition and continued to undergo the administration of chemotherapy in the oncology department according to the oncologist’s recommendations.

## 3. Review

To ensure a rigorous review, we conducted a literature search that encompassed multiple academic databases, including PubMed, Embase, the Cochrane Library, Web of Science, and Scopus. We found a total of 17 cases of *irinotecan* hypersensitivity reactions. The results are summarized in Table 3. 

## 4. Discussion

For oncology patients, chemotherapeutics are life-saving drugs. However, if a hypersensitivity reaction occurs, treatment is often stopped due to the distress that arises concerning the possibility of future reactions. The treating physician has to decide between switching to second-line therapy and manipulating the immune response through a desensitization protocol to induce tolerance to an essential drug.

Hypersensitivity reactions may occur during the administration of any chemotherapy agent, with some of them being more frequent triggers than others, and responses can range in severity from mild to severe, with the latter including anaphylaxis and even death. According to the class of the chemotherapy agent, there are different patterns in which a reaction can take place. For example, hypersensitivity reactions to platinum compounds usually develop after a few administrations, typically after four to six cycles, and the subsequent use of the same compound can trigger a more severe reaction. On the other hand, taxanes usually induce reactions during the first or second infusion and future administrations carry a lower risk of eliciting a reaction [1].

Since *irinotecan* hypersensitivity reactions are rare, their pattern has not been entirely described, although according to the currently published cases, their pattern of appearance seems to be similar to that of platinum compounds. Most of the recorded platinum compound-induced hypersensitivity reactions were IgE-mediated, and their skin test concentrations have been published [1]. The same mechanism has been proposed for *irinotecan*-induced hypersensitivity reactions, although the data are scarce.

All the reported cases, which total seventeen to date [7,8,9,10,11,15], involved immediate reactions, compatible with the diagnosis of anaphylaxis. Regarding the ones that were not detailed in the literature [7,11], we assumed that they also involved anaphylaxis because skin tests, followed by a desensitization protocol in one case [11], were performed. The intensity of the reactions ranged from moderate to severe, with cardiovascular and respiratory involvement.

Among all the reviewed reactions, skin test results are available for four patients [8,9,10,15], with all of them presenting positive results, confirming the IgE-mediated mechanism of the reactions. The first study to specify the concentrations used for skin testing with *irinotecan* is the one published by Alvarez-Cuesta et al. [7], which indicates a concentration of 20 mg/mL (undiluted drug) for prick tests, as well as 2 mg/mL and 20 mg/mL for intradermal tests. However, the authors do not mention the results obtained. The following articles that conducted skin tests report using the same drug concentrations. We also used the concentrations mentioned in the previous case reports and we obtained negative skin prick tests and a positive intradermal test at 2 mg/mL (1/10 of the maximum non-irritative concentration). That, therefore, validates the concentration already published and raises the hypothesis that *irinotecan*-induced hypersensitivity reactions may be IgE-mediated. This is also supported by the fact that the reaction developed at the beginning of the 11th cycle, suggesting that a previous sensitization reaction had occurred. In three previous cases, the patients also developed symptoms after several cycles [8,9,15]. The case reported by Andriollo et al. [10] depicts a reaction that occurred during the second administration of *irinotecan*, which was an IgE-mediated one, demonstrated by a positive intradermal test.

Following a chemotherapy agent-induced hypersensitivity reaction, the current guidelines mention the possibility of re-administration of the same drug with modified infusion rates under strict supervision, regardless of the underlying mechanism [1]. However, in cases of IgE-mediated reactions, it is known that this is not feasible, and a controlled desensitization protocol must be used instead. In our case, we demonstrated the IgE-mediated mechanism and we went for a rapid drug desensitization protocol that was well tolerated by the patient. The protocol was adapted from that of Castells et al. [14]; we started with a slower infusion rate and gradually increased until we reached a slightly higher infusion rate than in the original protocol during the last step of the protocol. The time interval between each step was respected and the total cumulative dose was adapted according to the dose needed, decided by the attending oncologist.

In six of the reported cases [8,9,10,11,15], the drug was re-administered using desensitization protocols, with all of them following the *rituximab* desensitization protocol described by M. Castells [14] and adapting the steps and premedication to each individual patient.

Effective collaboration between the attending oncologist and the allergist is crucial for determining the appropriate management of such a patient and the necessity for a desensitization protocol. In this case, a successful desensitization protocol was carried out due to the vital importance of continuing treatment with *irinotecan*.

In such cases, desensitization protocols for chemotherapy agents are useful procedures that can prolong the survival of oncology patients by providing immune tolerance to the culprit drug. The protocol may be adapted according to the particularities of the case and may be performed regardless of the results of skin tests. Still, in the case of discontinuously administered drugs, the protocol must be followed for every administration, as it only provides temporary tolerance.

Due to the lack of reported cases of *irinotecan* hypersensitivity, each published report adds valuable knowledge about its underlying mechanism, the usefulness of skin tests in its diagnosis, and its desensitization schedule.

There may be some possible limitations in this study. One of them is related to the small number of cases of hypersensitivity reactions to *irinotecan* and the even smaller number of skin test results and desensitization protocols for *irinotecan* published in the literature. This makes it difficult to generalize findings and form definite conclusions regarding the mechanisms of hypersensitivity reactions and the validation of certain desensitization protocols for *irinotecan*. Another limitation of the review is related to the omission of papers published in languages other than English, as well as research presented at various scientific events. The available data refer to case reports or case series. The largest case series, which comprises 11 patients, does not give details about the severity of the reactions nor about the occurrence of the reaction to the first dose, so the reviewed data comprise incomplete information.

Hypersensitivity reactions to *irinotecan* are rare; however, when they take place, they can severely limit treatment options in oncology patients. Given the important role of *irinotecan* in treating a variety of cancers, desensitization protocols can offer life-saving options for patients who have no other therapeutic alternative. By presenting information on the limited number of published cases of hypersensitivity reactions to *irinotecan* and the limited number of skin test results and desensitization protocols carried out, our study contributes to a better understanding of the mechanisms of hypersensitivity reactions to *irinotecan* and the safety profiles of the desensitization protocols used, thereby enhancing knowledge about the possible management of such cases.

Future research should focus on the assessment of hypersensitivity reactions to *irinotecan* in terms of mechanism, pattern of appearance, and clinical characteristics, as well as the standardization of a desensitization protocol for *irinotecan*, thereby contributing to a better understanding of hypersensitivity reactions to *irinotecan* and more appropriate management.

## 5. Conclusions

We identified a total of seventeen reported cases of hypersensitivity reactions to *irinotecan* in the literature, with ours being the eighteenth. Out of the available cases, four included skin test results, all of which presented positive results, confirming the IgE-mediated mechanism of the reactions. In six of the cases, desensitization protocols for *irinotecan* were carried out. For patients who have suffered a hypersensitivity reaction to *irinotecan* and for whom no other chemotherapy agent seems to have the same level of efficacy, a desensitization protocol for *irinotecan* may be the only option for administering it safely and thereby improving the patients’ prognosis.

## Figures and Tables

**Table 1:** Skin tests for *irinotecan* and their results.

*Irinotecan* Solution Concentration	0.2 mg/mL (1/100)	2 mg/mL (1/10)	20 mg/mL (1/1)
Skin prick test	Not performed	−	−
Intradermal test	−	+	Not performed

“−“—negative result; “+”—positive result.

**Table 2:** *Irinotecan* desensitization protocol adapted from Castells et al. [14].

Step	Solution	Administration Rate (mL/h)	Time (min)	Administered Volume (mL)	Administered Dose (mg)	Cumulative Administered Dose (mg)
1	1	1	15	0.25	0.0012	0.0012
2	1	2.5	15	0.625	0.003	0.0042
3	1	5	15	1.25	0.006	0.0102
4	1	10	15	2.5	0.012	0.0222
5	2	5	15	1.25	0.06	0.0822
6	2	10	15	2.5	0.12	0.2022
7	2	20	15	5	0.24	0.4422
8	2	40	15	10	0.48	0.9222
9	3	10	15	2.5	1.2	2.1222
10	3	20	15	5	2.4	4.5222
11	11	40	15	10	4.8	9.3222

**Table 3:** Reported cases of *irinotecan* hypersensitivity reactions.

Author, Year	Reported Number of Patients	Patient Age	Patient Sex	Neoplasm	History of *Irinotecan* Administration	Time of Reaction	Clinical Picture (as Described by Author)	Skin Test Results	Premedication	Desensitization Protocol
Alvarez-Cuesta et al., 2015 [7]	11	UNK	UNK	UNK	UNK	UNK	UNK	Concentrations used: 20 mg/mL for prick test, 2 and 20 mg/mL for ID test; results not available	N.A.	Not performed
Abu-Amna et al., 2015 [8]	1	39	F	Colorectal cancer	6 cycles well tolerated; re-administration after 1 year—1st cycle well tolerated	2nd cycle of 2nd regimen	Urticaria, flushing, itching, shortness of breath, oxygen desaturation, cough, wheezing, chest tightness, tachycardia, hypotension, nausea, vomiting, diarrhea	Marked sensitivity to skin test with *irinotecan* diluted 1:1000 (+), 1:100 (++), and 1:10 (++) *	Yes	12 steps, adapted from Castells [14]
Cubero et al., 2016 [9]	1	57	M	Low rectal neoplasm	4 cycles well tolerated; re-administration after 2 months	1st cycle of 2nd regimen	Lingual angioedema, generalized urticaria, desaturation, blurred vision	Positive ID test 20 mg/mL	Yes	12 steps, adapted from Castells [14]
Kendirlinan et al., 2019 [11]	1	35	F	Colorectal cancer	N.A.	1st cycle	Grade 2, moderate (not detailed)	Not performed	Yes	12 steps, Castells [14]
Andriollo et al., 2020 [10]	1	36	M	Pancreatic adenocarcinoma	1st administration well tolerated	2nd and 3rd administration	Dyspnea, labial and eyelid angioedema	Positive ID test (20 mg/mL)	Yes	12 steps, adapted from Castells [14]
Çakmak et al., 2021 [15]	2	UNK	UNK	Colorectal cancer Colorectal cancer	N.A. 1st cycle well tolerated	1st cycle 2nd cycle	Nausea, vomiting Nausea, vomiting, hypotension	Not performed Positive prick test (20 mg/mL) and positive ID test (2mg/mL)	Yes Yes	12 steps, Castells [14] 16 steps, Castells [14]

“N.A”—not applicable; “UNK”—unknown; ID—intradermal; * exact concentrations not mentioned.

## Data Availability

All the presented data are available upon request.

## References

[1] Pagani M., Bavbek S., Alvarez-Cuesta E., Berna Dursun A., Bonadonna P., Castells M., Cernadas J., Chiriac A., Sahar H., Madrigal-Burgaleta R. (2022). Hypersensitivity reactions to chemotherapy: An EAACI Position Paper. Allergy.

[2] Madrigal-Burgaleta R., Vazquez-Revuelta P., Marti-Garrido J., Lleonart-Bellfill R., Ali F.R., Alvarez-Cuesta E. (2021). Medical algorithm: Diagnosis and treatment of hypersensitivity reactions to cancer chemotherapy. Allergy.

[3] Bumbacea R.S., Ali S., Corcea S.L., Spiru L., Nitipir C., Strambu V., Bumbacea D. (2021). Omalizumab for successful chemotherapy desensitisation: What we know so far. Clin. Transl. Allergy.

[4] Reyhanoglu G., Smith T. (2023). Irinotecan. StatPearls [Internet].

[5] Bailly C. (2019). Irinotecan: 25 years of cancer treatment. Pharmacol Res..

[6] Kciuk M., Marciniak B., Kontek R. (2020). Irinotecan-Still an Important Player in Cancer Chemotherapy: A Comprehensive Overview. Int. J. Mol. Sci..

[7] Alvarez-Cuesta E., Madrigal-Burgaleta R., Angel-Pereira D., Ureña-Tavera A., Zamora-Verduga M., Lopez-Gonzalez P., Berges-Gimeno M.P. (2015). Delving into cornerstones of hypersensitivity to antineoplastic and biological agents: Value of diagnostic tools prior to desensitization. Allergy.

[8] Abu-Amna M., Hassoun G., Hadad S., Haim N., Bar-Sela G. (2015). Successful Desensitization Protocol for Hypersensitivity Reaction Caused by Irinotecan in a Patient With Metastatic Colorectal Cancer. Clin. Colorectal. Cancer.

[9] Cubero J.L., Escudero P., Yubero A., Millán P., Sagredo M.A., Colás C. (2016). Successful Desensitization to Irinotecan After Severe Hypersensitivity Reaction. J. Investig. Allergol. Clin. Immunol..

[10] Andriollo G., Urbani S., Buonomo A., Aruanno A., Nucera E. (2020). Rapid protocol for irinotecan desensitization: A case report and literature review. Allergo. J. Int..

[11] Kendirlinan R., Gümüşburun R., Çerçi P., Özbek E., Altıner S., Çelebi Sözener Z., Soyyiğit Ş., Aydın Ö., Bavbek S. (2019). Rapid Drug Desensitization with Chemotherapeutics (Platins, Taxanes, and Others): A Single-Center Retrospective Study. Int. Arch. Allergy Immunol..

[12] Castells M.C., Solensky R., Post T.W. (2020). Rapid Drug Desensitization for Immediate Hypersensitivity Reactions.

[13] Turner P.J., Ansotegui I.J., Campbell D.E., Cardona V., Carr S., Custovic A., Durham S., Ebisawa M., Geller M., Gonzalez-Estrada A. (2024). Updated grading system for systemic allergic reactions: Joint Statement of the World Allergy Organization Anaphylaxis Committee and Allergen Immunotherapy Committee. World Allergy Organ. J..

[14] Castells M.C., Tennant N.M., Sloane D.E., Hsu F.I., Barrett N.A., Hong D.I., Laidlaw T.M., Legere H.J., Nallamshetty S.N., Palis R.I. (2008). Hypersensitivity reactions to chemotherapy: Outcomes and safety of rapid desensitization in 413 cases. J. Allergy Clin. Immunol..

[15] Çakmak M.E., Kaya S.B., Can Bostan Ö., Öztürk Aktaş Ö., Damadoğlu E., Karakaya G., Kalyoncu A.F. (2022). Successful desensitization with chemotherapeutic drugs: A tertiary care center experience. Eur. Ann. Allergy Clin. Immunol..

